# Human papillomavirus (HPV) types among Alaska native women attending a colposcopy clinic in Anchorage, Alaska, 2009–2011

**DOI:** 10.1186/s13027-020-00283-x

**Published:** 2020-03-03

**Authors:** N. J. Murphy, L. R. Bulkow, M. Steinau, E. F. Dunne, E. Meites, L. E. Markowitz, E. R. Unger, T. W. Hennessy

**Affiliations:** 1 Southcentral Foundation, Anchorage, AK USA; 2 Arctic Investigations Program, Division of Preparedness and Emerging Infections, National Center for Emerging and Zoonotic Infectious Diseases, Centers for Disease Control and Prevention, Anchorage, AK USA; 3 Division of High Consequence Pathogens and Pathology, National Center for Emerging and Zoonotic Infectious Diseases, Centers for Disease Control and Prevention, Atlanta, GA USA; 4 Division of HIV/AIDS Prevention, National Center for HIV/AIDS, Viral Hepatitis, STD, and TB Prevention, Centers for Disease Control and Prevention, Atlanta, GA USA; 5 Division of Viral Diseases, National Center for Immunization and Respiratory Diseases, Centers for Disease Control and Prevention, Atlanta, GA USA

**Keywords:** Human papilloma virus, HPV 16, HPV 18, HPV vaccine, Cervical intraepithelial neoplasia, Squamous intraepithelial lesion, Cervical cancer, Cervical cytology

## Abstract

**Background:**

The first HPV vaccines licensed targeted two HPV types responsible for most cervical cancers. A 9-valent vaccine (9vHPV), targeting 5 additional types, was introduced in 2016 and is currently the only HPV vaccine available in the United States. Previous studies demonstrated high rates of HPV infection in Alaska Native (AN) women. We sought to measure prevalence of high risk HPV types in AN women undergoing colposcopy and to determine those preventable by vaccination.

**Methods:**

For this cross-sectional study, we recruited women who were undergoing colposcopy for clinical indications at Alaska Native Medical Center to obtain cervical brush biopsy samples. Specimens were shipped to Atlanta, Georgia for DNA extraction, HPV detection, and typing using L1 PCR with type-specific hybridization to detect 37 HPV types.

**Results:**

Four hundred eighty eight specimens from 489 women were tested. At least one HPV type was found in 458 (94%) specimens. Of 458 participants who were HPV positive, 332 (72%) had two or more types. At least one type targeted by 9vHPV was detected in 95% of participants with CIN 3 (21/22), 82% with CIN 2 (37/45), and 65% with CIN 1 (119/184). (*p* < 0.001) HPV 16 or 18 were detected in 77% (17/22) with CIN 3, 53% (24/45) with CIN 2, and 36% (67/184) with CIN 1. (*p* < 0.001).

**Conclusions:**

A substantial proportion of AN women attending colposcopy clinic had evidence of HPV 16/18 infection, as well as other high risk types targeted by 9vHPV. At least one 9vHPV type was detected in 62% of the participants overall, and 95% of participants with CIN3. AN women are expected to benefit from vaccination against HPV 16/18, and will have greater benefit from 9vHPV. Information from this study could be used to develop public health strategies to increase vaccine uptake, or to track HPV genotype prevalence over time.

## Background

Human papilloma virus (HPV) infection is common among young Alaska women and these infections cause many cases of cervical cancer among Alaska Native people [1–5]. In a 1988 study, genotypes 16/18/31/33/35 were detected in 234 cervical specimens (21%) from 1126 Alaska Native (AN) women seeking routine care and colposcopy or from population-based lists at the Alaska Native Medical Center (ANMC) in Anchorage, Alaska [2]. More than 95% of persons with HPV were found to have either 16/18 or 31/33/35. In a 1996 study at ANMC HPV 16 was found to be the HPV genotype most commonly associated with cervical intra-epithelial neoplasia (CIN) 2/3 [5].

The worldwide HPV prevalence in cervical carcinomas is 99.7% [6]; more than two-thirds of cervical cancers are associated with HPV 16 and 18 [7, 8]. A review of cervical cancer specimens collected from women during 1980–1989 found HPV 16 to be the most prevalent type in AN women, (77%), Greenland Native people, (81%), and Danish whites, (70%) [4]. A follow-up study of cervical cancer specimens collected during 1980–2007 found that 61.2% contained HPV types 16 or 18 [1]. While there has been a slight downward trend in cervical cancer in studies of circumpolar indigenous women from 1989 to 2008 [9], the age-standardized incidence rates among circumpolar Inuit women were twice those of circumpolar Dene women, 21.1 vs 10.0 per 100,000 [9].

In a 2000 study cervical cancer specimens from 53 Alaska Native people, 32 Greenland Native people and 34 Danish Caucasians from 1980 to 1989 were analyzed by polymerase chain (PCR) for HPV types 16, 18, 31, 33, and 45 [4]. Infections with multiple types were prevalent in AN (36.5%) but not in Greenland Native people (3.7%) or Danish Caucasians (6.9%). A 2013 study of 62 cases of invasive cervical cancer diagnosed in AN women aged 18 and above between 1980 and 2007 identified from the Alaska Native Tumor Registry were tested for HPV using Linear Array PCR [1]. One or more HPV type was detected in 91.9%. Sixty one percent of the cancer specimens contained HPV types 16 or 18, and 29% contained an oncogenic type other than type 16 or 18.

Vaccines against HPV types 16 and 18 have been in use since 2006 in the United States [10]. The quadrivalent HPV vaccine (Gardasil®, Merck and Co., Inc.) confers protection against types 16 and 18, which together cause approximately 70% of cervical cancer, and types 6 and 11 which are associated with 90% of genital warts. The Center for Disease Control and Prevention (CDC) Advisory Committee on Immunization Practices (ACIP) recommends HPV vaccine for routine use in U.S. girls and boys at age 11–12 years (or starting at age 9 years), through age 26 years for girls not previously vaccinated and through age 21 years for males not previously vaccinated. Quadrivalent HPV vaccine was made available through the State of Alaska Vaccine Distribution Program’s formulary (2006–2016) [11].

In February 2015 ACIP recommended 9 valent HPV vaccine (9vHPV) (Gardasil 9, Merck and Co., Inc.) as one of three HPV vaccines that can be used for routine vaccination. 9vHPV is a noninfectious, virus-like particle (VLP) vaccine [12]. Similar to quadrivalent HPV vaccine (4vHPV), 9vHPV contains HPV 6, 11, 16, and 18 VLPs. In addition, 9vHPV contains HPV 31, 33, 45, 52, and 58 VLPs [12]. The 9vHPV vaccine became available 4 years after our study period was initiated. Since late 2016, it is the only HPV vaccine being distributed in the United States.

A multi-agency Alaskan working group was established in 2006 to develop projects to improve HPV vaccine uptake and evaluate the impact on cervical cancers and other health outcomes. The group called *Statewide HPV Vaccine for Alaska Natives: Projects and Evaluations (SHAPE)* developed this project with the goal of establishing HPV genotype surveillance among a risk population. We sought to obtain a baseline of HPV prevalence that could be used in future evaluations to determine changes in HPV vaccine-type prevalence after vaccine introduction.

## Methods

The SHAPE group undertook a cross-sectional study to determine HPV genotypes in AN women undergoing colposcopy for abnormal cervical screening results at ANMC in 2009. ANMC, located in Anchorage, Alaska, is a tertiary care center for the Alaska Native Health Consortium which also serves as the primary and secondary health facility for approximately 140,000 Alaska Native and American Indians people.

All non-pregnant Alaska Native Health System beneficiaries 18 years old or older undergoing indicated colposcopy at the ANMC from October 1, 2009 – September 30, 2011 were eligible to participate. The colposcopy was indicated based on the American Society for Colposcopy and Cervical Pathology cervical cancer screening consensus algorithms. Exclusion criteria included age less than 18 or pregnancy. A routine urine pregnancy test was done prior to colposcopy. Basic demographic data, tobacco use, and vaccination status were obtained though the electronic medical record at the time of the colposcopy. There was no information collected for women who refused enrollment.

After informed consent, a clinician-collected cervical sample was obtained at the time of pelvic examination. After visualization of the cervical os, the sample was taken with a Digene Cervical Sampler brush (Qiagen Inc., Valencia, CA). The brush was placed in specimen transport medium (STM, Qiagen Inc.) and stored in the ANMC colposcopy refrigerator, then transported on ice to the CDC Arctic Investigations Program in Anchorage, Alaska USA and was frozen. Specimens were batched and shipped on dry ice to CDC in Atlanta, Georgia, USA for extraction, HPV detection, and typing using the Roche linear array test as previously described [13]. Specimens were labeled with study identification and testing was performed without personal identifiers in accordance with the Alaska Area Institutional Review Board and the Alaska Native Tribal Health Consortium and Southcentral Foundation board recommendations. Diagnostic categories were assigned based on the highest grade noted in the clinical pathologist’s histologic diagnosis.

The de-identified data were analyzed with proportions of patients with HPV types compared using a nonparametric test for trend across ordered groups. *P*-values less than 0.05 were considered statistically significant and all tests were 2-sided. HPV types 16, 18, 31, 33, 35, 39, 45, 51, 52, 56, 58, 59, and 68 were considered the 13 high risk types [14]. Analysis was conducted in STATA version 10.

## Results

Five hundred and twenty specimens from 489 women were collected and tested. In the 31 women who were tested twice, the first study specimen was included, unless no biopsy obtained at that time (*n* = 4), then the second specimen was used. One additional woman was excluded because she did not have a cervix. Four hundred and eighty eight specimens were included in the final analysis.

Self-reported ethnic groups of the sample included: Inuit/Yupik 238 (49%), Aleut 73(15%), Athabascan /Tlingit /Haida 149 (31%), and other 28 (5%). The mean age of the participants was 29.7 years. The largest age group was less than 25 years old, 40%, followed by 25–29 years (25%). (Table 1) Current tobacco use was reported by 246 (51%) of the 484 in which tobacco use status was documented.

**Table 1:** Study Population Demographics and Outcomes, Anchorage, Alaska 2009–2011 (*N* = 488 total specimens)

	N (%)	Mean (Standard Deviation)
Age Group		
< 25 yrs	194 (40%)	29.7 yrs. (9.4 yrs)
25–29 yrs	124 (25%)	
30–34 yrs	78 (16%)	
35–39 yrs	34 (7%)	
40–44 yrs	20 (4%)	
45–49 yrs	13 (3%)	
≥ 50 yrs	25 (5%)	
Ethnic Group		
Eskimo	238 (49%)	
Indian	149 (31%)	
Aleut	73 (15%)	
Mixed	17 (3%)	
Non-Native	9 (2%)	
Non-specific Native	2 (0.4%)	
Tobacco use, ever	246/484 (51%)	
Current tobacco user	225/471 (48%)	
Number of HPV vaccine doses		
0	440 (90%)	
1	18 (3.7%)	
2	9 (1.8%)	
3	20 (4.1%)	
4	1 (0.2%)	
Ectocervical biopsy done	438 (90%)	
Endocervical curettage done	402 (82%)	
Loop Electrosurgical Excision Procedure done	42 (8.6%)	
Cytology Referral Diagnosis		
Atypical Squamous Cells of Undetermined Significance	231 (47%)	
Low-grade Squamous Intraepithelial Lesion	162 (33%)	
High-grade Squamous Intraepithelial Lesion	46 (9%)	
Atypical Squamous Cells cannot exclude High-Grade Squamous Intraepithelial cells	25 (5%)	
Unknown	24 (5%)	
Final Pathology Diagnosis		
CIN* 3	22 (5%)	
CIN 2	45 (9%)	
CIN 1	184 (38%)	
Metaplasia/Inflammation	135 (28%)	
Other**	46 (9%)	
Insufficient/No biopsy	55 (11%)	
Number of HPV types in HPV positive specimens		
1	126 (28%)	2.6 (SD = 1.57)
2	126 (28%)	Median = 2
3	94 (21%)	
4	55 (12%)	
5	31 (7%)	
6+	26 (6%)	
Number of 9-valent HPV vaccine types present		
0	184 (38%)	0.88 (SD = 0.87)
1	205 (42%)	
2	76 (16%)	
3	19 (4%)	
4	4 (0.8%)	

*CIN Cervical intraepithelial neoplasia

** Other = Atrophy, Atypia, Negative

Forty eight (10%) had received any HPV vaccine and only twenty one (4.3%) had completed a three dose series at the time of the colposcopy. No significant difference was found in HPV type distribution or diagnostic category between participants who received any vaccine doses and those who received no vaccine. There was no significant difference in HPV type distribution or diagnostic category between tobacco using and non-tobacco using participants.

The most common cytology referral diagnosis was atypical squamous cells of undetermined significance 231 (47.3%), followed by low grade squamous intraepithelial lesion (162; 33.2%), high grade squamous intraepithelial lesion (46; 9.4%), atypical squamous cells cannot exclude high-grade squamous intraepithelial lesion (25; 5.1%), and unknown (24, 4.9%).

An ectocervical biopsy was obtained from 438 (90%) women and an endocervical curettage was performed in 402 (82%). There were 42 (8.6%) loop electrosurgical excision procedure (LEEP) specimens. CIN 3 was found in 22 (4.5%) specimens; CIN 2 (45; 9.5%), CIN 1 (184; 37.7%), metaplasia (135; 27.6%), other (46; 9.0%), no biopsy (6; 1.2%), and insufficient (49; 10.0%).

**Fig. 1:**
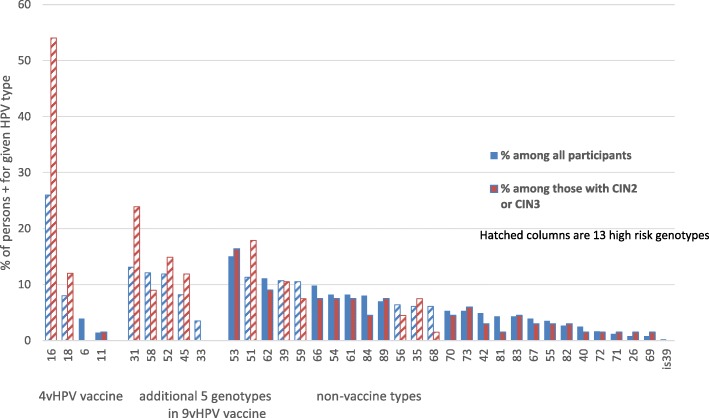
Participants with HPV detected according to genotype, among all participants and those with Cervical Intraepithelial Neoplasia (CIN) 2 or 3, Anchorage, Alaska, 2009–2011. 13 High risk types include: 16, 18, 31, 33, 35, 39, 45, 51, 52, 56, 58, and 59

One or more of the 37 HPV types were found in 458 (94%) specimens. Of the 458 participants who were HPV positive, 332 (72%) had two or more types. Among those with any HPV detected, the mean number of HPV types per participant was 2.6. (Table 1) Three hundred and four specimens were positive for one or more of the 9vHPV types (62%); 171 for 4vHPV genotypes (35%) including; 153 for HPV 16/18 (31%); and 392 for any of the 13 high risk types (80%). (Fig. 1).

Of the 9vHPV high-risk types, type 16 was found in 127 (26%) of all participants; type 31 in 64 (13%); type 58 in 59 (12%); type 52 in 58 (12%); type 45 in 40 (8%); type 18 in 39 (8%); and type 33 in 15 (3%). (Fig. 1).

There was a mean of 0.88 9vHPV types per participant. A single 9vHPV type was detected in 205 (42%) AN women. 76 (16%) had 2 types, 19 (4%) 3 types, and 4 (0.8%) 4 types. There was a mean of 0.88 9vHPV types per participant. (Table 1).

There was no significant difference in HPV genotype distribution or diagnostic category by vaccination history or by history of tobacco use. (Table 1).

One or more of the 9vHPV types was detected in 95% with CIN 3 (21/22), 82% with CIN 2 (37/45), and 65% with CIN 1 (119/184) (*p* < 0.001). (Table 2) HPV 16 or 18 was detected in 77% (17/22) with CIN 3, 53% (24/45) with CIN 2, and 36% (67/184) with CIN 1. (*p* < 0.001).

**Table 2:** HPV genotypes detected according to colposcopy diagnosis groups (includes colposcopy and endocervical curettage diagnosis), Anchorage, Alaska 2009–2011)

	Diagnosis						
	CIN 3	CIN 2	CIN 1	Other*	N/A**	P-value	
	*N* = 22	*N* = 45	*N* = 184	*N* = 182	*N* = 55	Trend ***	CIN 3/2 vs CIN1/Other
HPV 16/18 (any)	17 (77%)	24 (53%)	57 (31%)	40 (22%)	15 (27%)	**< 0.001**	**< 0.001**
HPV 6/11 (any)	0 (0%)	1 (2%)	14 (8%)	10 (5%)	1 (2%)	0.071	0.151
13 HR HPV types^	21 (95%)	44 (98%)	160 (87%)	124 (68%)	43 (78%)	**0.043**	**< 0.001**
9vHPV 9 types	21 (95%)	37 (82%)	119 (65%)	95 (52%)	32 (58%)	**< 0.001**	**< 0.001**

*Other includes: metaplasia, inflammation, atrophy, atypia, and negative

**N/A includes: insufficient and no biopsy

***Trend for increasing prevalence of HPV types from CIN 1- to CIN 3

^HPV 13 high risk types: 16, 18, 31, 33, 35, 39, 45, 51, 52, 56, 58, 59, 68

## Discussion

In this study of HPV types in mostly unvaccinated women presenting for colposcopy at the Alaska Native Medical Center, we found 35% had any of the 4vHPV types and 62% had any of the 9vHPV types. The most commonly detected type was HPV 16. Detection of 16/18 and any of the 9vHPV types was more common with higher severity of CIN. Any 9vHPV type was detected in 62% of participants overall, and 95% of participants with CIN3. Since 96% of our participants were not fully vaccinated, these results represent a near baseline HPV genotype prevalence before widespread impact of the HPV vaccines among women attending colposcopy clinic.

The detection of HPV 16/18 in women with CIN2+ was higher in AN women than has been reported in areas in the United States outside of Alaska [15]. HPV 16/18 was found in 61.1% of women with CIN 2+ in AN in this study compared to 50.1% reported in CIN2+ lesions in the five catchment areas in the HPV-IMPACT project [15]. The detection of HPV 16/18 among AN was more similar to the non-Hispanic whites, (56.4%), than to the racial/ethnic minorities in the HPV-IMPACT project, (41.8–45.9%) [15].

The distribution of HPV types in this AN group was similar to the pattern reported in American Indian women in the Northern Plains [3, 16]. In a study of cervical samples collected from 287 women attending a Northern Plains American Indian reservation general outpatient clinic, 61 women (21.2%) tested positive for HPV. Among all HPV-positive women, 41% had multiple HPV types. Schmidt-Grimminger reported HPV infections among AI women showed a wider variety and very different pattern of HPV types than non-Native women. This included a higher prevalence of multiple HPV infections (19% [95% CI = 26–38] vs. 7% [95% CI = 4–11]; *p* = 0.001) [16].

Of the 458 AN participants who were HPV positive, 332 (72%) had two or more types, with an mean of 2.6 HPV types per specimen. This is a similar pattern to previous findings in AN women in 1980–1989 in which multiple types were found in 43% of AN women [4] and 21% of AN women in 1988–1990 [2]. This was in contrast to 4% multiple types found in Greenland Native women and 7% Danish Caucasians in 1980–1989 [4].

This study has some important limitations. The participating women were a convenience sample of all patients presenting to colposcopy clinic. However, the clinical staff estimated they enrolled approximately 95% of eligible patients, minimizing selection bias. Thus, these results are likely generalizable to Alaska Native women with abnormal cytology who receive care at Alaska Native Medical Center. The proportion of women who had received any doses of HPV vaccine series was very low and there was no difference in HPV genotype distribution by vaccine status. So, although this group was not entirely vaccine naïve, these data effectively represent the HPV genotypes in a population with abnormal cervical screening prior to use of HPV vaccine.

This study provides data which may help to plan future evaluations of HPV genotype prevalence and vaccine impact among this clinical population. For example, 532 participants 18 years or older would be needed to detect a change from a baseline prevalence of HPV 16/18 of 31 to 25%, using an alpha = 0.05, beta = 0.10. One hundred fifty seven participants 18 years or older would be needed to detect a decreased rate to 20% for HPV 16/18 positive. For comparison, assuming that the HPV type changes would be more rapidly detected in participants < 25 years of age, only 272 participants would be necessary to detect a change from a baseline rate of 34% for HPV 16/18 to 25%; whereas 106 participants < 25 years of age would be needed to detect a decrease of HPV 16/18 from the baseline of 34 to 20%. From these calculations, focusing on younger women would improve efficiency and reduce the impact of the study on clinical services. Consideration could also be given to using recruitment strategies other than continuous enrollment, such as the first week of the month.

## Conclusion

A substantial proportion of AN women attending colposcopy clinic have HPV 16/18, as well as the other high risk types in 9vHPV - 31/33/45/52/59. While any of the 9vHPV types were detected in 62% of the participants overall, this increased to 95% of participants with CIN3. AN women would be expected to benefit from vaccination against HPV 16/18, and would be expected to have greater benefit from 9vHPV. In 2014, the CDC reported that 81.2% AN adolescent females aged 13–17 years received one dose of HPV vaccine, while only 58.3% received all three HPV vaccine doses as recommended by ACIP at that time [17]. Because providers and patients are interested in local data, this study could be used to create public health strategies to increase vaccine uptake in Alaska in the future [18].

## Data Availability

The datasets used and/or analyzed during the current study are available from the corresponding author on reasonable request.

## Abbreviations

ACIP: Advisory Committee on Immunization Practices, CDC
AN: Alaska Native
ANMC: Alaska Native Medical Center
CDC: Center for Disease Control and Prevention
CIN: Cervical intraepithelial neoplasia
HPV: Human papilloma virus
LEEP: Loop electrosurgical excision procedure
PCR: Polymerase chain reaction
SHAPE: Statewide HPV Vaccine for Alaska Natives: Projects and Evaluations
VLP: Virus-like particle
